# Bictegravir and Metformin Drug-Drug Interaction in People with Human Immunodeficiency Virus (HIV)

**DOI:** 10.3390/idr15030024

**Published:** 2023-04-25

**Authors:** Anne M. Masich, Lindsey Thompson, Patricia P. Fulco

**Affiliations:** 1 Department of Pharmacy, Virginia Commonwealth University Health, Richmond, VA 23298, USApatricia.fulco@vcuhealth.org (P.P.F.); 2 Department of Pharmacotherapy and Outcome Sciences, School of Pharmacy, Virginia Commonwealth University, Richmond, VA 23298, USA; 3 Department of Internal Medicine, Division of Infectious Diseases, Virginia Commonwealth University Health, Richmond, VA 23298, USA

**Keywords:** bictegravir, metformin, HIV, diabetes, drug-drug interaction, people with human immunodeficiency virus

## Abstract

A drug-drug interaction (DDI) exists between bictegravir and metformin. Bictegravir inhibits renal organic cation transporter-2, leading to increased metformin plasma concentrations. The objective of this analysis was to evaluate the clinical implications of concomitant bictegravir and metformin administration. This was a retrospective, single-center, descriptive analysis evaluating people with human immunodeficiency virus (PWH) concurrently prescribed bictegravir and metformin between February 2018–June 2020. PWH lost to follow-up or non-adherent were excluded. Data collection included: hemoglobin A1C (HgbA1C), HIV RNA viral load, CD4 cell count, serum creatinine, and lactate. Adverse drug reactions (ADRs) were assessed by provider-documented, patient-reported symptoms of gastrointestinal (GI) intolerance and hypoglycemia. Metformin dose adjustments and discontinuations were recorded. Fifty-three PWH were included (116 screened; 63 excluded). GI intolerance was reported in three PWH (5.7%). There were no documented episodes of hypoglycemia or lactic acidosis. Five PWH had metformin dose reductions (N = 3 for unspecified reasons; N = 1 for GI intolerance) or discontinuation (N = 1 unrelated to ADRs). Both diabetes and HIV control improved (HgbA1C decreased by 0.7% with virologic control in 95% of PWH). Minimal ADRs were reported in PWH receiving concurrent metformin and bictegravir. Prescribers should be aware of this potential interaction; however, no empiric metformin total daily dose adjustment appears necessary.

## 1. Introduction

Type 2 diabetes mellitus (T2DM) is becoming an increasingly prevalent comorbid condition in people with human immunodeficiency virus (PWH). A recent cross-sectional study from 2015 identified a relative risk of T2DM in PWH of 2.4 compared to the general population and a prevalence of 15.1%, which increased from 6.8% in 2005 [1]. Traditional risk factors, including increased age and obesity, contribute to the development of T2DM among PWH. However, PWH may have higher T2DM prevalence at younger ages and in the absence of obesity [2]. HIV infection is associated with an increased risk of insulin resistance, and the initiation of antiretroviral therapy (ART) may result in metabolic alterations, weight gain, and T2DM [3,4]. 

Recent data suggest integrase strand transfer inhibitors (INSTIs) and tenofovir alafenamide (TAF) result in significant weight gain, followed by emerging reports of new diabetes diagnoses [5,6,7]. An analysis of the Federal Food and Drug Administration (FDA) Adverse Event Reporting System linked INSTIs [bictegravir and dolutegravir] to hyperglycemia or new-onset T2DM [5,6]. Nearly 50% of these reports were patient-initiated with only 23% by providers. In an additional retrospective analysis of ART-naïve adults initiated on treatment between 2007–2017, a three-fold higher risk of new-onset T2DM after 18 months resulted when comparing PWH on INSTI-containing regimens to non-INSTI-based therapy [5,8,9]. In rare cases, bictegravir is associated with accelerated hyperglycemia and diabetic ketoacidosis (DKA) [10]. A recent case series described three PWH who developed DKA within weeks to months after bictegravir initiation, despite only slightly elevated hemoglobin A1C (HgBA1C) at baseline. Following the discontinuation of bictegravir, all three PWH had significant reductions in insulin requirements and HgBA1C [10]. These reports demonstrate the increased risk of PWH developing chronic diseases, such as diabetes; however, the longer life expectancy with the newest antiretroviral drugs may also be a confounding factor associated with the development of age-associated comorbidities [3,5,6]. 

According to the American Diabetes Association, metformin remains a preferred initial pharmacologic agent for the treatment of T2DM with an assessment of concurrent morbidities [11]. Current HIV treatment guidelines recommend INSTIs as initial ART for most PWH [8,9]. With the increasing incidence of T2DM in PWH and initial regimens, including INSTIs, the potential for concomitant use with metformin is high. Two INSTIs, bictegravir and dolutegravir, inhibit renal organic cation transporter-2 (OCT2) and multidrug and toxin extrusion-1 (MATE-1) transporter in the kidneys in vitro, inhibiting the tubular secretion of creatinine in the kidneys [12,13]. This inhibition results in increasing plasma concentrations of OCT2 and MATE-1 substrates (e.g., metformin), potentially leading to adverse drug reactions (ADRs). FDA labeling for both bictegravir and dolutegravir indicates increased metformin plasma concentrations by 28% and 66%, respectively, when administered concomitantly [12,13]. It is recommended to initiate metformin at the lowest dose with concurrent dolutegravir, but no bictegravir dose adjustments are necessary [8]. The clinical significance of these interactions remains uncertain [12,13]. A 2017 case series (N = 19) evaluating the clinical implications of concomitant dolutegravir-metformin reported six PWH with ADRs, including gastrointestinal (GI) distress and hypoglycemic symptoms resulting in five metformin dose reductions and/or discontinuations [14]. The results of a 2017 retrospective analysis assessing concomitant dolutegravir-metformin reported no cases of hypoglycemia or lactic acidosis as a result of the interaction [15]. A case of hyperlactatemia due to the co-administration of dolutegravir and metformin is additionally reported [16]. The conclusion of this case report has been challenged based on the timing to lactate normalization and the dolutegravir-metformin elimination half-lives [17]. 

Currently, there are limited data evaluating the drug-drug interaction (DDI) between bictegravir and metformin. The objective of this analysis was to determine the clinical implications of concomitant bictegravir and metformin administration in PWH.

## 2. Materials and Methods

### 2.1. Study Design

This was a retrospective, single-center descriptive analysis evaluating all adult PWH receiving care in a health-system-based infectious diseases clinic with concurrent prescriptions for bictegravir and metformin between February 2018–June 2020. PWH who did not receive concomitant bictegravir and metformin, who were lost to follow-up after initiation, or who were non-adherent to bictegravir or metformin therapy were excluded as determined by medication fill history or documentation of poor retention in care by the provider. 

### 2.2. Data Collection

Electronic medical records were reviewed beginning at bictegravir-metformin initiation, at three, and six-to-twelve months after concomitant therapy. Baseline demographics, including previous dolutegravir use, were collected. Metformin-related ADRs, including patient-reported GI upset or intolerance, symptoms of hypoglycemia, and metformin total daily dose (TDD) adjustments or discontinuations, were assessed. HgbA1C, metformin dosing regimens, and administration of other antihyperglycemic agents were used to evaluate T2DM management. HIV control was determined by HIV RNA viral load (VL) and immunologic response (CD4 cell count). Weight change from baseline was additionally recorded. The laboratory safety analysis included alterations in serum lactate (if measured) and serum creatinine (SCr). 

### 2.3. Statistical Analysis

Data were analyzed using descriptive statistics. Discrete data were presented as the number of PWH (n) and the proportion of the study population (%). Continuous data were presented as the median and interquartile range (IQR). To provide additional comparisons and insights, PWH were stratified based on previous dolutegravir exposure vs. INSTI naïve as a dolutegravir-based multi-tablet regimen is often simplified to the fixed-dose single bictegravir-based pill for therapy simplification. 

## 3. Results

A total of 116 PWH were identified with prescriptions for both bictegravir and metformin, with 53 PWH meeting the inclusion criteria. PWH were excluded for the following: no concurrent use of bictegravir-metformin (N = 16), lost to follow-up (N = 22), and medication nonadherence (N = 25). Fifteen PWH received a dolutegravir-containing ART regimen directly prior to bictegravir use. Baseline demographics are described in Table 1 and stratified by prior dolutegravir use. The median baseline HgbA1C was above the therapeutic goal for the entire sample (7.5%). The metformin TDD was higher in INSTI naïve PWH compared to those with previous dolutegravir use (1000–2000 mg metformin TDD vs. 500–1000 mg, respectively). INSTI naïve PWH were prescribed more additional oral antihyperglycemic agents compared to those previously prescribed dolutegravir (sodium-glucose cotransport-2 inhibitors: 8% vs. 0%; sulfonylureas: 18% vs. 7%; dipeptidyl peptidase 4 inhibitors: 26% vs. 7%). More PWH with prior dolutegravir use were prescribed insulin compared to the INSTI naïve group (47% vs. 16%). 

Few PWH experienced ADRs with concurrent bictegravir-metformin use: three experienced GI upset or intolerance (5.7%) with no reported hypoglycemic episodes or lactic acidosis (Table 2). Of note, one person who experienced GI intolerance was previously prescribed dolutegravir. Metformin TDD was modified to alleviate GI intolerance (N = 1), to optimize T2DM therapy (N = 7), and for unspecified reasons (N = 3). Metformin TDD reductions in INSTI naïve PWH (N = 3) did not correlate with reported GI intolerances. Metformin TDD was decreased by 500 mg in the person with GI intolerance who had previous dolutegravir use. Metformin was discontinued for one person in response to a normalized HgbA1C.

T2DM control improved with a reduced HgbA1C of 6.9% and 6.8% at three and at six-to-twelve months, respectively, after bictegravir-metformin initiation. The immunologic status did not change significantly throughout the study period, with HIV virologic control (defined as VL <50 copies/mL) improving from 75% at baseline to 87% and 95% at three and six-to-twelve months, respectively. At three months, a median increase of 0.05–0.17 mg/mL in the SCr resulted as expected by OCT2 inhibition with no further significant change in SCr after six-to-twelve months of bictegravir treatment. The total population had a median weight gain of 10.8 kg from baseline to 6–12 months. INSTI naïve PWH had a larger median weight gain of 10 kg compared to 7.7 kg in PWH with prior dolutegravir use. However, 40% of PWH demonstrated weight loss during the study period.

## 4. Discussion

The results of our study in PWH receiving concurrent bictegravir and metformin demonstrated minimal clinical implications of the DDI between bictegravir and metformin despite an increase in metformin area under the plasma concentration (AUC) from reduced renal elimination [18]. Few ADRs were observed, with GI intolerance occurring in 5% of persons with no reports of hypoglycemia or hyperlactatemia. Metformin dose adjustments only occurred in two people (N = 1, GI intolerance; N = 1, normalized HgbA1C) as a result of ADRs or improved control. A recent study by Cattaneo et al. in PWH with T2DM (N = 20) on bictegravir and metformin similarly reported no hypoglycemic or lactic acidosis events in PWH with normal renal function [19]. 

Pharmacokinetic data with concurrent bictegravir-metformin administration demonstrate an increased metformin plasma total drug exposure by renal OCT2/MATE-1 inhibition [18]. In this Phase 1 placebo-controlled crossover study, 32 healthy volunteers received bictegravir/emtricitabine/TAF or placebo for nine days. On day four, metformin was administered for four days. Metformin AUC increased by 39% [% geometric mean ratio (90% CI): 139 (131–148)] with decreased renal clearance by 31%. Metformin pharmacodynamic response, evaluated by plasma glucose, was not significantly different between the groups (*p* > 0.05) [18]. 

For our total study population and when stratified by prior dolutegravir use, T2DM control improved over the follow-up period. Most of the PWH were on metformin for months to years prior to bictegravir initiation. It is possible that the bictegravir-metformin DDI improved HgbA1C and may be beneficial for T2DM control. However, concomitant bictegravir-metformin did not result in any clinically relevant effects on fasting plasma glucose or HgbA1C over a one-year period in the Cattaneo et al. study, but stratification based on dolutegravir use was not analyzed [19]. The clinical effect of bictegravir-metformin on improving T2DM control requires further evaluation in a larger sample population.

PWH without prior dolutegravir use were primarily maintained with oral antihyperglycemics alone, while a larger portion of PWH in the prior dolutegravir group required insulin. While we are limited by the retrospective data collection, the difference between the two groups may be reflective of more advanced T2DM due to longer exposure to HIV and ART in PWH with prior dolutegravir use, as indicated by the older age. This is consistent with previously published data in PWH suggesting drug-disease interactions [including ART-related metabolic disturbances (weight gain)] resulting in poor T2DM control [5,10,20]. 

Our total population had a median weight gain of approximately 11 kg by the end of the study period (40% lost weight). This effect on weight supports recent data demonstrating weight gain associated with INSTI use [20]. In this pooled analysis of weight gain in eight clinical trials, the 96-week least squares mean weight gain in PWH receiving INSTI-containing ART was 3.24 kg [95% CI, 3.02–3.46]. Bictegravir and dolutegravir had similar weight gain (bictegravir: 4.24 kg [95% CI, 3.71–4.78]; dolutegravir: 4.07 kg [95% CI, 3.51–4.62]) [20]. The majority of the weight gain occurred in the first 48 weeks. Of note, 30.2% of PWH included in this analysis lost weight. The mechanism by which INSTIs affect weight is not fully known but may be attributed to improved GI tolerability compared to older ART regimens and faster virologic control leading to a return-to-health phenomenon, in which the PWH weight returns to or exceeds the pre-illness baseline. 

Limitations to this study include the retrospective cohort study design. Subjective information, including reported ADRs, may be incompletely documented, and complete laboratory data was absent for a large proportion of included PWH. This could be attributed to current HIV treatment guidelines recommending an extension of laboratory monitoring to 6-month intervals for virologically suppressed PWH [8]. The inclusion of PWH with previous dolutegravir use prior to initiating bictegravir decreased the bictegravir alone sample size. Separate analyses were conducted for those with and without prior dolutegravir in order to identify and highlight any impact on the results. These authors feel the inclusion of PWH with prior dolutegravir use is a strength of this study as this more closely reflects clinical practice when bictegravir is prescribed for ART simplification.

## 5. Conclusions

Although bictegravir increases metformin plasma concentrations, the results of our study demonstrate empiric metformin dose reduction with concomitant bictegravir administration appears unnecessary to minimize ADRs. We recommend providers concurrently prescribing bictegravir and metformin should be aware of this interaction and continue regular monitoring for associated ADRs, especially in PWH with renal impairment due to a potential increased risk for lactic acidosis. While concurrent bictegravir-metformin administration may be beneficial for T2DM control, more studies are needed. 

## Figures and Tables

**Table 1:** Baseline characteristics [median (IQR)].

	Total Population (N = 53)	No Previous DTG (N = 38)	Previous DTG (N = 15)
Age, yr	56.0 (49–60)	55.5 (49–60)	57.0 (49–63)
Female, N (%)	17 (30.9)	12 (31.6)	5 (33.3)
Race, N (%)			
Black White Other	34 (62) 17 (31) 2 (4)	24 (63) 12 (32) 2 (5)	10 (67) 5 (33) 0 (0)
Weight, kg	92.5 (82–113)	93.3 (79–113)	92.5 (88–111)
Metformin TDD, N (%)			
500 mg 750 mg 1000 mg 1500 mg 2000 mg	11 (20) 1 (2) 25 (45) 2 (4) 14 (25)	8 (21) 1 (3) 15 (39) 1 (3) 13 (34)	3 (20) 0 (0) 10 (67) 1 (7) 1 (7)
Other antidiabetic agents, N (%) ^a^			
Insulin Oral agents ^b^ Injectables ^c^	13 (24) 23 (42) 5 (9)	6 (16) 20 (53) 3 (8)	7 (47) 3 (20) 2 (13)
HgbA1C, %	7.5 (6.5–9.8)	7.7 (6.5–10)	7.3 (6.6–8.6)
SCr, mg/dL	0.91 (0.76–1.11)	0.89 (0.73–1.09)	1.01 (0.82–1.18)
CD4 cell count, cells/mm^3^	728 (446–1028)	728 (460–1042)	540 (442–684)
HIV RNA VL < 50 copies/mL, N (%)	39 (71)	28 (74)	11 (73)

DTG: dolutegravir; HgbA1C: glycated hemoglobin; SCr: serum creatinine; TDD: total daily dose; VL: viral load. ^a^ Some patients prescribed multiple agents (DTG naïve N = 8; previous DTG N = 2) ^b^ Sodium-glucose cotransport-2 inhibitors (DTG naïve N = 3), sulfonylureas (DTG naïve N = 7; previous DTG N = 1), dipeptidyl peptidase 4 inhibitors (DTG naïve N = 10; previous DTG N = 1), thiazolidinediones (DTG naïve N = 1; previous DTG N = 1). ^c^ Glucagon-like peptide 1 agonists (DTG naïve N = 5; previous DTG N = 2).

**Table 2:** Primary and Secondary Outcome Results.

	Total Population (N = 53)		No Previous DTG (N = 38)		Previous DTG (N = 15)	
Patient-Reported ADRs [N(%)]						
Gastrointestinal intolerance	3 (5.7)		2 (5.3)		1 (6.7)	
Hypoglycemia	0 (0)		0 (0)		0 (0)	
Lactic acidosis	0 (0)		0 (0)		0 (0)	
Metformin TDD Adjustments [N(%)]						
Dose increase	6 (11.3)		5 (13.2)		1 (6.7)	
Dose decrease	4 (7.5)		3 (7.9) *		1 (6.7) ^&^	
Discontinuation	1 (1.9)		0 (0)		1 (6.7) ^#^	
Laboratory Data [median(IQR)]						
	3 mo	6–12 mo	3 mo	6–12 mo	3 mo	6–12 mo
CD4 cell count, cells/mm^3^	750 (501–983)	687 (455–1055)	750 (549–1008)	687 (540–1118)	442 (408–612)	459 (419–887)
HIV RNA VL < 50 copies/mL, N (%)	26 (87)	38 (95)	19 (83)	24 (92)	7 (100)	14 (100)
HgbA1C, %	6.9 (6.4–8.1)	6.8 (6.1–7.7)	6.8 (6.4–7.7)	6.8 (6–7.8)	7.2 (6.3–8.2)	6.6 (6.2–7.2)
SCr, mg/dL	1.06 (0.93–1.16)	1.05 (0.80–1.20)	1.06 (0.86–1.15)	1.08 (0.80–1.24)	1.06 (0.98–1.19)	0.99 (0.83–1.15)
Weight, kg	95.0 (81–115)	103.3 (79–115)	99.3 (79–113)	103.3 (81–115)	91.8 (86–104)	100.1 (93–113)

ADRs: adverse drug reactions; DTG: dolutegravir; HgbA1C: glycated hemoglobin; SCr: serum creatinine; TDD: total daily dose; VL: viral load. * Unspecified patient or provider reasons. ^#^ Dose change per primary care provider for well-controlled HgbA1C. ^&^ Associated with GI upset intolerance.

## Data Availability

The data presented in this study are available on request from the corresponding author, e.g., privacy or ethical. The data are not publicly available due to private health information restrictions within the Virginia Commonwealth Health system.

## References

[1] Duncan A.D., Goff L.M., Peters B.S. (2018). Type 2 diabetes prevalence and its risk factors in HIV: A cross-sectional study. PLoS ONE.

[2] Hernandez-Romieu A.C., Garg S., Rosenberg E.S., Thompson-Paul A.M., Skarbinski J. (2017). Is diabetes prevalence higher among HIV-infected individuals compared with the general population? Evidence from MMP and NHANES 2009–2010. BMJ Open Diabetes Res. Care.

[3] Noubissi E.C., Katte J.C., Sobngwi E. (2018). Diabetes and HIV. Curr. Diab. Rep..

[4] Venter W.D.F., Sokhela S., Simmons B., Moorhouse M., Fairlie L., Mashabane N., Serenata C., Akpomiuemie G., Masenya M., Qavi A. (2020). Dolutegravir with emtricitabine and tenofovir alafenamide or tenofovir disoproxil fumarate versus efavirenz, emtricitabine, and tenofovir disoproxil fumarate for initial treatment of HIV-1 infection (ADVANCE): Week 96 results from a randomised, phase 3, non-inferiority trial. Lancet HIV.

[5] Asundi A., Olson A., Jiang W., Patel S., White L.F., Sagar M., Lin N.H. (2020). 946. Risk factors and metabolic implications of integrase inhibitor associated weight gain. Open Forum. Infect. Dis..

[6] Murray M.M., Harpe S.E. (2020). 106. META-INSTI: Metabolic adverse events following integrase strand transfer inhibitor administration in spontaneous adverse event reports. Open Forum. Infect. Dis..

[7] McComsey G.A., Emond B., Shah A., Bookgart B.K., Rossi C., Milbers K., Lafeuille M.-H., Donga P. (2022). Association Between Weight Gain and the Incidence of Cardiometabolic Conditions Among People Living with HIV-1 at High Risk of Weight Gain Initiated on Antiretroviral Therapy. Infect. Dis. Ther..

[8] Panel on Antiretroviral Guidelines for Adults and Adolescents. Guidelines for the Use of Antiretroviral Agents in Adults and Adolescents with HIV. Department of Health and Human Services. Published May 2022. https://clinicalinfo.hiv.gov/en/guidelines/adult-and-adolescent-arv/what-start-initial-combination-regimens-antiretroviral-naïve.

[9] World Health Organisation (WHO) Policy Brief: Update of Recommendations on First-and Second-Line Antiretroviral Regimens. WHO Libr. Cat. Data..

[10] Nolan N.S., Adamson S., Reeds D., O’Halloran J.A. (2021). Bictegravir-Based Antiretroviral Therapy-Associated Accelerated Hyperglycemia and Diabetes Mellitus. Open Forum. Infect. Dis..

[11] American Diabetes Association (2022). Introduction: Standards of Medical Care in Diabetes—2022. Diabetes Care.

[12] (2019). Biktarvy (Bictegravir, Emtricitabine, Tenofovir Alafenamide Fumarate) [Package Insert]. Food and Drug Administration. https://www.accessdata.fda.gov/drugsatfda_docs/label/2019/210251s006lbl.pdf.

[13] (2013). Tivicay (Research Triangle Park, NC: ViiV Healthcare) [Package Insert]. Food and Drug Administration. https://www.google.com.hk/url?sa=t&rct=j&q=&esrc=s&source=web&cd=&ved=2ahUKEwia2Pe_wcT-AhXUlWoFHeU_CiMQFnoECA0QAQ&url=https%3A%2F%2Fwww.accessdata.fda.gov%2Fdrugsatfda_docs%2Flabel%2F2013%2F204790lbl.pdf&usg=AOvVaw2ByzLR-xJIgrPdHZOXV3v4.

[14] Masich A., Badowski M.E., Liedtke M.D., Fulco P.P. (2017). Evaluation of the concurrent use of dolutegravir and metformin in human immunodeficiency virus-infected patients. Int. J. STD AIDS.

[15] Gervasoni C., Minisci D., Clementi E., Rizzardini G., Cattaneo D. (2017). How relevant is the interaction between dolutegravir and metformin in real life?. JAIDS J. Acquir. Immune Defic. Syndr..

[16] Naccarato M., Yoong D., Fong I.W. (2017). Dolutegravir and metformin: A case of hyperlactatemia. AIDS.

[17] Cattaneo D., Resnati C., Rizzardini G., Gervasoni C. (2018). Dolutegravir and metformin: A clinically relevant or just a pharmacokinetic interaction?. AIDS.

[18] Custodio J., West S., Yu A., Martin H., Graham H., Quirk E., Kearney B. (2017). Lack of clinically relevant effect of bictegravir (BIC, B) on metformin (MET) pharmacokinetics (PK) and pharmacodynamics (PD). Open Forum. Infect. Dis..

[19] Cattaneo D., Formenti T., Minisci D., Casalini G., Meraviglia P., Gervasoni C. (2021). Lack of clinically relevant interactions between bictegravir and metformin in persons with diabetes and HIV. J. Antimicrob. Chemother..

[20] Sax P.E., Erlandson K.M., Lake J.E., Maaomsey G.A., Orkin C., Esser S., Brown T.T., Rockstroh J.K., Wei X., Carter C.C. (2020). Weight gain following initiation of antiretroviral therapy: Risk factors in randomized comparative clinical trials. Clin. Infect. Dis..

